# Polysaccharides from *Polygonatum cyrtonema* Hua Reduce Depression-Like Behavior in Mice by Inhibiting Oxidative Stress-Calpain-1-NLRP3 Signaling Axis

**DOI:** 10.1155/2022/2566917

**Published:** 2022-04-20

**Authors:** Fengming Shen, Pan Xie, Congting Li, Zhijuan Bian, Xuncui Wang, Daiyin Peng, Guoqi Zhu

**Affiliations:** ^1^Key Laboratory of Xin'an Medicine, The Ministry of Education, Anhui University of Chinese Medicine, Hefei, Anhui 230012, China; ^2^Key Laboratory of Molecular Biology (Brain Diseases), Anhui University of Chinese Medicine, Hefei, Anhui 230012, China; ^3^Anhui Province Key Laboratory of Chinese Medical Formula, Anhui University of Chinese Medicine, Hefei, Anhui 230012, China

## Abstract

Polysaccharides from *Polygonatum cyrtonema* Hua (PSP) exert antioxidant, anti-inflammatory, and antidepressant effects. Production of reactive oxygen species (ROS) and activation of the calpain system and the NOD-like receptor protein 3 (NLRP3) inflammasome are closely related to the pathogenesis of depression. However, the relationships among those pathways and the protective effects of PSP have not been characterized. In this study, lipopolysaccharide (LPS) and chronic unpredictable mild stress- (CUMS-) induced depression models were used to evaluate the protective mechanisms of PSP against depression. ROS levels were measured in HT-22 cells using flow cytometry. Brain tissues were collected to determine the levels of oxidation-related indicators and inflammatory cytokines. The protein levels of calpain-1, calpain-2, calpastatin, phosphatase and Tensin Homolog deleted on Chromosome 10 (PTEN), suprachiasmatic nucleus circadian oscillatory protein (SCOP), nuclear factor-erythroid factor 2-related factor 2 (Nrf2), heme oxygenase-1 (HO-1), NLRP3, apoptosis-associated speck-like protein (ASC), caspase-1, cleaved-caspase-1, ionized calcium binding adapter molecule 1 (Iba1), phosphorylation of extracellular signal-regulated kinase (p-ERK), nuclear factor-kappa B (NF-*κ*B), interleukin-1*β* (IL-1*β*), and glial fibrillary acidic protein (GFAP) were measured using western blotting or immunofluorescence. In cellular experiments, we showed that PSP attenuated LPS-induced production of ROS in HT-22 cells. In animal experiments, we found that LPS increased the expression of calpain-1, NLRP3, ASC, caspase-1, cleaved-caspase-1, Iba1, p-ERK, NF-*κ*B, and GFAP and reduced the expression of calpastatin, PTEN, SCOP, and Nrf2. Administration of PSP reversed these changes. N-Acetyl-L-cysteine (NAC) administration also inhibited oxidative stress and activation of the calpain system and the NLRP3 inflammasome. Furthermore, PSP, calpeptin, MCC950 (a selective NLRP3 inflammasome inhibitor), and NAC reduced LPS-induced proinflammatory cytokine release. We also showed that PSP prevented CUMS-induced changes in the calpain system and the Nrf2 and NLRP3 signaling pathways and reduced depression-like behavior. These results indicate that PSP exerts antidepressant effects through regulation of the oxidative stress-calpain-1-NLRP3 signaling axis.

## 1. Introduction

According to the World Health Organization (WHO) in 2018, more than 300 million people worldwide suffer from depression and more than 800,000 have committed suicide because of depression [1]. The WHO has listed depression as a particularly impactful disability [2]. Most individuals with depression, especially those with severe depression, do not fully recover and are likely to experience recurrence [3]. Drugs currently used for treatment of depression suffer from low efficiency, numerous side effects, and susceptibility to relapse upon discontinuation of drug treatment. Therefore, it is critical to explore the mechanisms of depression to aid in development of more effective antidepressants.

Clinical studies have shown that oxidative markers, including reactive oxygen and nitrogen species such as 8-hydroxy-2′-deoxyguanosine and F2-isoprostanes, were elevated in the serum of patients with depression [4, 5]. Moreover, nuclear factor-erythroid factor 2-related factor 2- (Nrf2-) dependent antioxidative activity was proposed as a therapeutic target for depression [6]. In addition to oxidative stress, neuroinflammation has been considered as a key player in the pathogenesis of depression. Patients with depression have higher plasma levels of proinflammatory cytokines [7]. NOD-like receptor protein 3 (NLRP3) plays an important role in regulating immune function and inflammatory responses [8]. In a clinical study, serum NLRP3 levels were increased in patients with major depressive disorder [9]. Inhibition of NLRP3 has been shown to ameliorate lipopolysaccharide- (LPS-) induced depression-like behaviors [10]. Although oxidative stress, neuroinflammation, and activation of NLRP3 are associated with the development of depression, the relationship among these factors has not been characterized.

Calpains, a family of calcium-dependent proteases, are activated by high concentrations of calcium [11]. There are two major calpain subtypes in the brain, calpain-1 and calpain-2, that play important roles in learning and memory and are associated with neuronal damage [12, 13]. We previously reported that activation of calpain was closely related to the occurrence of neuroinflammation during development of depression-like behaviors. Inhibition of calpain reduced degradation of its substrates, which resulted in inhibition of downstream inflammatory pathway-related proteins and improvement of depression-like behavior [14]. Increased oxidative stress and excitotoxicity may promote abnormal activation of calpain [15]. Based on these findings, calpains may act as intermediaries between reactive oxygen species (ROS) and NLRP3-dependent inflammation and may contribute to pathogenesis of depression.

Plant polysaccharide is an important active ingredient of medicinal plants and exerts antioxidant, anti-inflammatory, and immunomodulatory effects [16]. A variety of plant polysaccharides have shown significant antidepressant effects. For an example, lentinan can induce significant antidepressant-like effects by activating the prefrontal Dectin-1/*α*-amino-3-hydroxy-5-methyl-4-isoxazole propionic acid receptor signaling pathway [17]. *Polygonatum sibiricum* is a medicine and food and has been used in a number of traditional Chinese prescriptions for the treatment of depression [18]. The main components of PS are polysaccharides, steroidal saponins, flavonoids, and other active components. *Polygonum sibiricum* polysaccharide, one of the main active components of *Polygonatum sibiricum*, exerts antioxidant, anti-inflammatory, and antitumor effects; enhances immunity; and can be used to treat diabetes mellitus and atherosclerosis [19]. In a previous study, we reported that depression-like behavior could be reduced by polysaccharides from *Polygonatum cyrtonema* Hua (PSP) with a molecular weight distribution of 6~14 kDa [20]. However, the potential mechanisms have not been reported.

To determine the interplay among oxidative stress, inflammation, NLRP3, and calpains in the development of depression-like behavior, we conducted this study using LPS and chronic unpredictable mild stress (CUMS) models. In addition, we investigated the mechanisms of action of PSP in depression. This study supported the use of PS to treat depression.

## 2. Materials and Methods

### 2.1. Animals and Drugs

Male C57BL/6J mice (2 months old, weighing 20–25 g) were purchased from the Experimental Animal Center of Anhui Medical University [SCXK (Wan) 2016-0009]. The mice were kept in a room with constant temperature (22 ± 2°C), appropriate relative humidity (45–65%), and well-ventilated conditions. A 12 h light/dark cycle was maintained, and the mice were allowed free access to food and water. The mice were adaptively fed for one week before the experiments. The experimental procedures were approved by the Ethics Committee of Anhui University of Chinese Medicine, Hefei, China (Approval No. AHUCM-mouse-20210205).

Calpeptin was purchased from Selleckchem (S7396, HPLC > 97%, USA). N-Acetyl-L-cysteine (NAC) was purchased from Beyotime Biotechnology (S0077, HPLC > 97%, Shanghai, China). MCC950 sodium, a selective NLRP3 inflammasome inhibitor, was purchased from Selleckchem (S7809, HPLC > 99.57%).

### 2.2. Preparation and Identification of PSP

PSP was extracted from dried rhizomes of *Polygonatum cyrtonema* Hua (Anhui Senfeng Agricultural Comprehensive Development Co., Ltd., Hefei, China) as previously described [20]. PSP was obtained via water extraction and alcohol precipitation followed by vacuum freeze drying. The purity of PSP was confirmed using a UV spectrophotometer. Finally, PSP ranging from 6 kDa to 14 kDa was dialyzed at room temperature for 48 h and concentrated to a solid-to-liquid ratio of 1 : 1 using a rotary evaporator. After alcohol precipitation, the solution was kept at -4°C overnight. The upper layer was removed, and the down layer was frozen at -20°C, followed by vacuum freeze drying. The PSP power was used in the pharmacological experiments.

### 2.3. Measurement of the Antioxidant Capacity of PSP

Ferric-reducing antioxidant power (FRAP) working solution and FeSO_4_ solutions at different concentrations (0.15, 0.3, 0.6, 0.9, 1.2, and 1.5 mM) were prepared. Then, 180 *μ*l of FRAP solution was added to each well of the 96-well plate. Five milliliters of each FeSO_4_ standard solution was added to the indicated wells. Different concentrations of PSP or vitamin C (VC) solution were added to the sample detection wells. After incubation at 37°C for 5 min, the absorbance was measured at 593 nm. FRAP values (mM) for different concentrations of PSP and VC were calculated using the standard curve, and the FRAP values were used to represent the total antioxidant capacities of VC and PSP.

### 2.4. Measurement of ROS Level in HT-22 Cells

Hippocampal neurons (HT-22 cells) in the logarithmic growth phase were trypsinized, then seeded in six-well plates. The cells were divided into a control group, an LPS group, and LPS+PSP (100, 200, and 400 *μ*g/ml) groups. The wells in the control group and the LPS group contained medium with 1% fetal bovine serum (FBS). The wells in the LPS+PSP groups contained 1% FBS with 100, 200, or 400 *μ*g/ml PSP for 2 h. Then, the cells in the LPS group and the LPS+PSP groups were cultured with 20 *μ*g/ml LPS for 24 h. The cells were collected and suspended in diluted dichloro-dihydro-fluorescein diacetate (DCFH-DA) at cell densities from 1 million to 20 million cells per milliliter. The cells were then incubated in a 37°C incubator for 20 min and washed three times with serum-free cell culture medium and then analyzed using flow cytometry (FC500, Beckman). ROS levels were analyzed using FlowJo software.

### 2.5. LPS Model and Treatments

#### 2.5.1. Experiment 1

**Figure 1:**
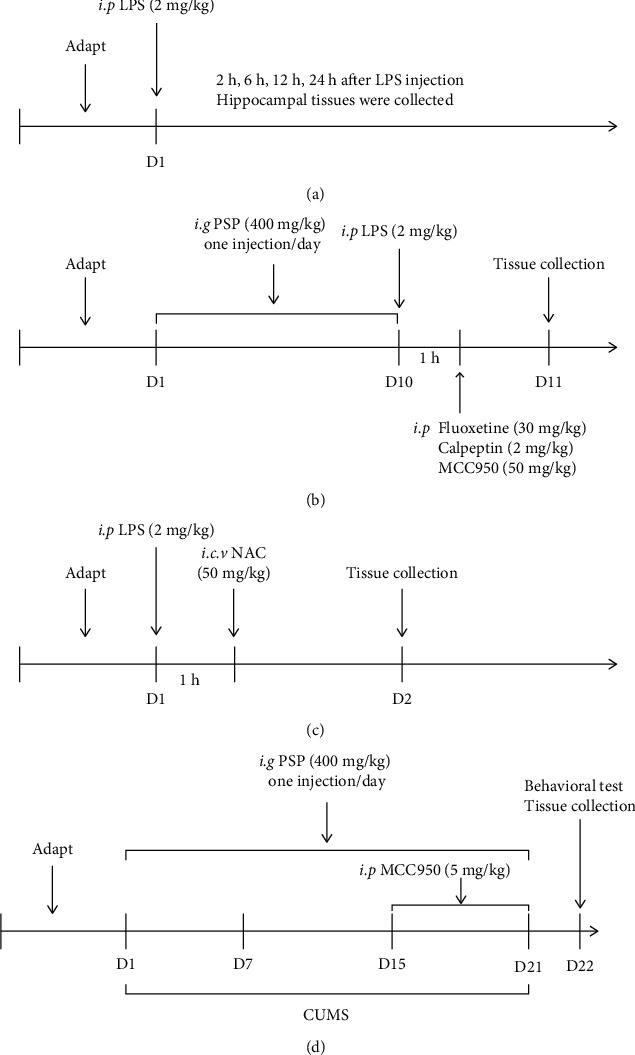
Schematic designs of this study. (a) shows the schematic design of experiment 1. (b) shows the schematic design of experiment 2. (c) shows the schematic design of experiment 3. (d) shows the schematic design of the CUMS experiment.

After 1 week of adaptation, the mice were injected with 2 mg/kg LPS in saline to establish a depression model as previously described [21]. The mice were randomly divided into five groups (*N* = 6): control, 2 h post-LPS, 6 h post-LPS, 12 h post-LPS, and 24 h post-LPS. At different time points after the LPS injection, the mice were anesthetized with 5% isoflurane and sacrificed via decapitation, and the hippocampi were collected (Figure 1(a)).

#### 2.5.2. Experiment 2

The mice were randomly divided into six groups (*N* = 6): control, LPS, LPS+PSP, LPS+fluoxetine, LPS+calpeptin, and LPS+MCC. After 1 week of adaptation, the mice were given 400 mg/kg PSP dissolved in distilled water via intragastric administration for 10 days, according to a previous study [20]. The other groups were given equal volumes of water. After 10 days, the mice in the LPS, LPS+fluoxetine, and LPS+calpeptin groups were given intraperitoneal injections of LPS. The LPS+fluoxetine group was given 30 mg/kg fluoxetine via intraperitoneal injection 1 h after LPS injection. The dose of fluoxetine was selected based on a previous publication [22]. The mice in the LPS+calpeptin group were given 2 mg/kg calpeptin via intraperitoneal injection 1 h after LPS injection. Calpeptin was dissolved in dimethyl sulfoxide and further diluted in saline as previously described [23]. The mice in the LPS+MCC group were given 50 mg/kg MCC950 dissolved in saline via intraperitoneal injection 1 h after LPS injection [24]. Twenty-four hours after LPS injection, the animals were anesthetized with 5% isoflurane and decapitated, and the hippocampi were collected (Figure 1(b)).

#### 2.5.3. Experiment 3

The mice were randomly divided into three groups (*N* = 6): control, LPS, and LPS+NAC. After 1 week of adaptation, the mice were given LPS via intraperitoneal injection. The mice in the LPS+NAC group were given a 3 *μ*l (2.5 mg/ml in saline) intraventricular injection (−0.8 mm AP, 0 mm ML, −2.0 mm DV to Bregma) of NAC (50 mg/kg) 1 h after LPS injection [25]. Twenty-four hours after LPS injection, the animals were anesthetized with 5% isoflurane and decapitated, and the hippocampi were collected (Figure 1(c)).

### 2.6. Establishment of CUMS Model and Treatments

The mice were randomly divided into four groups (*N* = 7): control, CUMS, CUMS+PSP, and CUMS+MCC. After 1 week of adaptation, a series of stimuli were selected to induce depression-like behaviors (food deprivation for 24 h, water deprivation for 24 h, restraint stress for 2 h, tail-clipping for 5 min, noise stress for 3 h, wet bedding for 24 h, bedding deprivation for 12 h, heat stress at 45°C for 5 min, cold stress at 4°C for 5 min, cage tilting for 12 h, cage shaking for 10 min, electrical stimulation, and inversion of the light/dark cycle). Two stimuli were randomly selected each day for a 21-day period. From the first day of modeling, the mice in the CUMS+PSP group were administered PSP intragastrically for 21 days at a dose of 400 mg/kg. This dose was selected based on a previous study [20]. From the 15th day, the mice in the CUMS+MCC group were given 5 mg/kg MCC950 via intraperitoneal injection for 7 days [26]. During CUMS, the body weight was measured once per week. Behavioral tests were performed after modeling. After the behavioral tests, the animals were anesthetized with 5% isoflurane and decapitated, and brain tissues were collected (Figure 1(d)).

### 2.7. Sucrose Preference Test (SPT)

The sucrose preference test was performed weekly during CUMS. In the first 24 h, each mouse was given two bottles of 1% sucrose in water. In the second 24 h, each mouse was given a bottle of water and a bottle of 1% sucrose. The amount of sucrose water consumed by each mouse was measured after 24 h.

### 2.8. Tail Suspension Test (TST)

The TST was performed and data were analyzed using SuperMaze software (Shanghai Xinruan Information Technology Co. Ltd., Shanghai China) as previously described [20].

### 2.9. Forced Swimming Test (FST)

The mice were placed in water (25°C) and allowed to swim for 6 min. The time of immobility during the last 4 min of the test was recorded.

### 2.10. Open Field Test (OFT)

The OFT was performed in a quiet environment as previously described [20], and the total distance travelled was calculated.

### 2.11. Measurement of Tissue Malondialdehyde (MDA) and Superoxide Dismutase (SOD)

The hippocampi were harvested to determine SOD and MDA levels. The hippocampus was weighed, extracting solutions from the SOD assay kit (BC0025, Solarbio, Beijing, China) or MDA assay kit (BC0175, Solarbio) were added, and the samples were homogenized on ice. The supernatants were placed on ice and analyzed per the manufacturer's instructions. The absorbance of each sample was measured at the indicated wavelengths (SOD: 560 nm; MDA: 600 nm, 532 nm, and 450 nm), and SOD and MDA levels were calculated based on a prepared standard curve.

### 2.12. Measurement of Inflammatory Cytokines

The hippocampi were harvested to determine the levels of interleukin-1*β* (IL-1*β*) and tumor necrosis factor-*α* (TNF-*α*) (JL18442 and JL10484, Jianglai, Shanghai, China). After reaction, the absorbance was measured at 450 nm using a microplate reader (1510, Thermo Scientific), and the levels of IL-1*β* and TNF-*α* were calculated according to the standard curve.

### 2.13. Western Blotting

The hippocampal tissues were homogenized with protein lysate (RIPA: PMSF = 100 : 1). Twenty-five micrograms of protein from each sample was separated via sodium dodecyl sulfate polyacrylamide gel electrophoresis (12% gels) for 50 min at 120 V. The separated proteins were transferred to nitrocellulose membranes for 2 h at 200 mA. The membranes were blocked with 5% fat-free milk for 2 h at room temperature, then were incubated with primary antibodies against calpain-1 (1 : 1,000, ProteinTech), calpain-2 (1 : 1,000, ProteinTech), calpastatin (1 : 1,000, ProteinTech), suprachiasmatic nucleus circadian oscillatory protein (SCOP) (1 : 500, Santa Cruz), phosphatase and Tensin Homolog deleted on Chromosome 10 (PTEN) (1 : 1,000, CST), Nrf2 (1 : 1,000, ZEN BIO), heme oxygenase-1 (HO-1) (1 : 1,000, ZEN BIO), NLRP3 (1 : 1,000, CST), apoptosis-associated speck-like protein (ASC) (1 : 1,000, ZEN BIO), caspase-1 (1 : 1,000, ProteinTech), cleaved-caspase-1 (1 : 1,000, CST), glial fibrillary acidic protein (GFAP) (1 : 1,000, Bioss), extracellular signal-regulated kinase (ERK) (1 : 1,000, CST), p-ERK (1 : 1,000, CST), and nuclear factor-kappa B (NF-*κ*B) (1 : 1,000, CST) at 4°C overnight. The membranes were washed three times with PBST (1 : 2,000 Tween-20: PBS), then incubated with horseradish peroxidase-conjugated goat antirabbit or antimouse IgG for 2 h at room temperature. The membranes were developed using ECL exposure solution, and the optical densities of the bands were analyzed using ImageJ software.

### 2.14. Immunofluorescence

Whole brains of mice were fixed in 4% formaldehyde for 48 h and dehydrated in 30% sucrose at 4°C for 48 h, then sectioned into 20 *μ*m thick slices. The slices were blocked in 0.1 M PBS containing 10% goat serum and 0.4% Triton X-100 for 1 h and incubated overnight at 4°C with an Nrf2 antibody (1 : 200, ZEN BIO). The slices were washed in 0.1 M PBS (15 min 3 times), then incubated for 2 h with FITC-labeled antirabbit IgG (1 : 100, ZSGB-BIO). During the final 10 min, DAPI (4′,6-diamidino-2-phenylindole) was added to stain the nuclei. The slices were visualized using an FV1000 Olympus laser confocal scanning microscope (Olympus, Tokyo, Japan). The Nrf2^+^ cell numbers were counted using ImageJ software.

### 2.15. Statistical Analyses

Data are presented as the mean ± standard error of mean (SEM) and analyzed using GraphPad Prism 8.0 software (GraphPad Inc., San Diego, CA, USA). *P* < 0.05 was considered statistically significant. Independent-sample *t*-tests or a one-way ANOVA followed by the Bonferroni test was used to compare differences between groups.

## 3. Results

### 3.1. Extraction, Identification, and Antioxidant Capacity of PSP

We extracted and isolated PSP with a molecular weight range of 6–14 kDa as previously described [20] and performed qualitative and quantitative analyses. Imaging analysis showed the reticular surface of the polysaccharide, and the energy spectrum indicated that its main elements were carbon (47.77%) and oxygen (30.31%). The results of ultraperformance liquid chromatography analysis showed that PSP was mainly comprised of arabinose, glucose, glucuronic acid, galactose, galacturonic acid, mannose, rhamnose, and ribose (sFig. 1).

**Figure 2:**
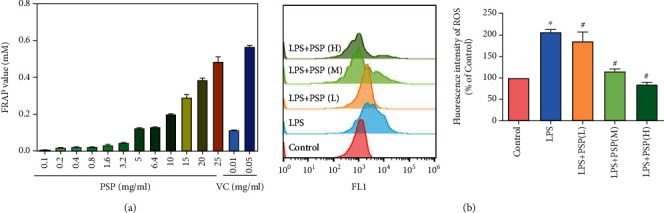
The antioxidant capacity of PSP. (a) shows the FRAP values of PSP and VC. (b) shows the ROS levels in HT-22 cells. (h, m, l) represent PSP at concentrations of 100, 200, and 400 *μ*g/ml, respectively. Data are presented as the mean ± SEM. ^∗^*P* < 0.05 vs. control, ^#^*P* < 0.05 vs. LPS group.

We measured the FRAP values of different concentrations of PSP and VC using the FRAP method. The results showed that the total antioxidant capacities of a 6.4 mg/ml PSP solution and of a 0.01 mg/ml VC solution were equivalent, and the total antioxidant activity of PSP was concentration-dependent (Figure 2(a)). The results of flow cytometry analysis showed that the fluorescence intensity of DCFH-DA in the LPS-treated HT-22 cells was significantly higher than that in the control group. Compared with the model group, the fluorescence intensity of DCFH-DA in the PSP group was significantly lower (Figure 2(b)). These results indicated that PSP inhibited LPS-induced production of cytoplasmic ROS in HT-22 cells. In our previous study, we also showed that PSP attenuated LPS-induced depression-like behaviors [20] and oxidative stress in the hippocampus of LPS-treated mice (sFig. 2, 3).

### 3.2. PSP Prevented LPS-Induced Expression of Calpain-1 and Degradation of Calpain-1-Dependent Substrates

**Figure 3:**
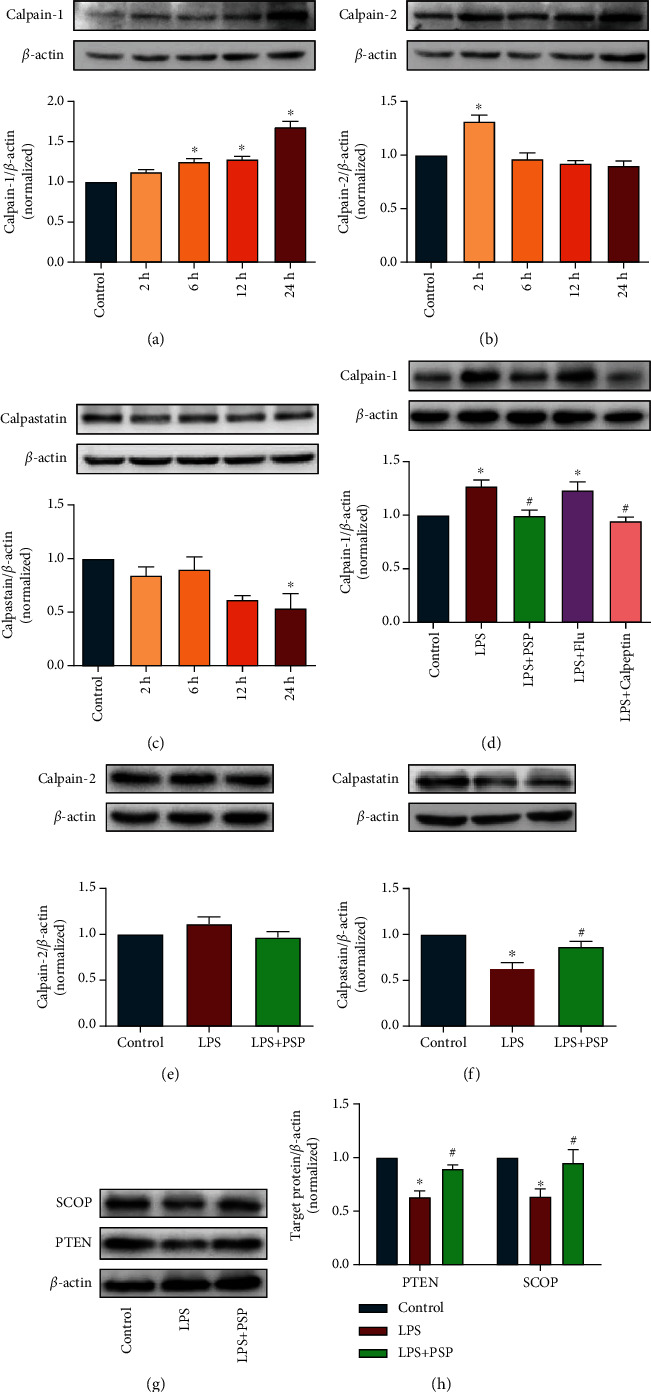
PSP inhibited LPS-induced expression of calpain-1 and degradation of its substrates. (a–c) show the representative blots of calpain-1, calpain-2, calpastatin, and quantification data for calpain-1/*β*-actin, calpain-2/*β*-actin, and calpastatin/*β*-actin in experiment 1. (d–f) show representative blots of calpain-1, calpain-2, calpastatin, and quantification data for calpain-1/*β*-actin, calpain-2/*β*-actin, and calpastatin/*β*-actin. (g) shows representative blots of SCOP and PTEN. (h) shows the quantification data for SCOP/*β*-actin and PTEN/*β*-actin. Data are presented as the mean ± SEM. ^∗^*P* < 0.05 vs. control, ^#^*P* < 0.05 vs. LPS group.

The expression of calpain-1 in the hippocampus increased from 2 to 24 h after LPS injection. At 24 h after LPS injection, calpain-1 expression was significantly higher than that in the control group (Figure 3(a), *P* < 0.05). The expression of calpain-2 was significantly increased at 2 h after LPS injection, then decreased to baseline (Figure 3(b)). The expression of calpastatin was decreased in response to LPS and was significantly lower at 24 h after LPS injection (Figure 3(c) vs. control, *P* < 0.05).

The effects of PSP were evaluated at 24 h after LPS injection. Treatment with 400 mg/kg PSP prevented LPS-induced increases in calpain-1 expression (Figure 3(d), *P* < 0.05). However, the expression of calpain-1 did not differ between the LPS and LPS+fluoxetine groups. We previously showed that a calpain inhibitor and fluoxetine could both prevent LPS-induced depression-like behaviors [14, 20] but had different effects on calpain-1 expression. Treatment with PSP did not affect calpain-2 expression (Figure 3(e), *P* > 0.05). Furthermore, treatment with PSP significantly inhibited the LPS-mediated decrease in calpastatin expression (Figure 3(f), *P* < 0.05).

We further evaluated the level of calpain substrates PTEN and SCOP. The results showed that the levels of PTEN and SCOP were significantly lower after LPS injection than those in the control group (Figure 3(g)). In contrast, the expression levels of PTEN and SCOP in the LPS+PSP group were significantly higher than those in the LPS group (Figures 3(g) and 3(h), *P* < 0.05). These results showed that LPS induced calpain-1 expression and triggered degradation of the calpain-dependent substrates PTEN and SCOP, and PSP prevented those abnormalities.

### 3.3. PSP Mitigated LPS-Mediated Decreases in Nrf2 Protein Expression

**Figure 4:**
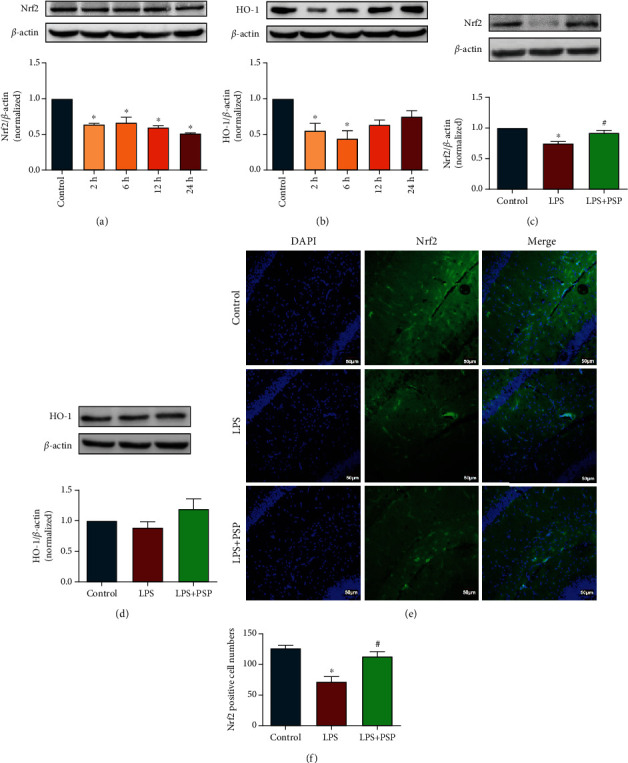
PSP alleviated LPS-induced oxidative damage by regulating the Nrf2 signaling pathway. (a, b) show representative blots of Nrf2, HO-1, and quantification data for Nrf2/*β*-actin and HO-1/*β*-actin after LPS treatment. (c, d) show representative blots of Nrf2 and HO-1 and quantification data for Nrf2/*β*-actin and HO-1/*β*-actin after PSP treatment. (e) shows immunofluorescence images of Nrf2 and DAPI. (f) shows quantitative measurements of Nrf2^+^ cells. Data are presented as the mean ± SEM. ^∗^*P* < 0.05 vs. control, ^#^P < 0.05 vs. LPS group.

The expression of Nrf2 in the hippocampus was decreased at 2, 6, 12, and 24 h after LPS injection. The expression of Nrf2 was significantly lower than that in the control group 24 h post-LPS injection (Figure 4(a), *P* < 0.05). The expression of HO-1 was significantly lower at 2 h and 6 h after LPS injection, then returned to baseline level (Figure 4(b), *P* < 0.05).

The effects of PSP on the Nrf2 signaling pathway were evaluated 24 h after LPS injection. The expression of Nrf2 was significantly higher in the LPS+PSP group than in the LPS group (Figure 4(c), *P* < 0.05). By contrast, the expression of HO-1 was comparable among the three groups (Figure 4(d), *P* > 0.05). We also detected Nrf2 expression using immunofluorescence. The expression of Nrf2 in the CA1 region of the hippocampus was significantly decreased following LPS injection. Treatment with PSP significantly ameliorated the LPS-mediated decrease in Nrf2 expression in CA1 (Figures 4(e) and 4(f), *P* < 0.05). Those results suggested that PSP could reduce LPS-induced oxidative damage through regulation of the Nrf2 pathway.

### 3.4. PSP Inhibited LPS-Induced Activation of the NLRP3 Inflammasome Signaling Pathway and Glial Cell Activation

**Figure 5:**
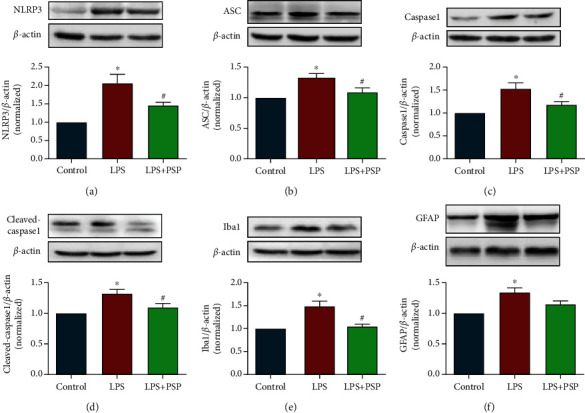
PSP inhibited LPS-induced NLRP3 pathway activation and glial cell activation. (a–d) show representative blots of NLRP3, ASC, caspase-1, and cleaved-caspase-1 and quantification data for NLRP3/*β*-actin, ASC/*β*-actin, caspase-1/*β*-actin, and cleaved-caspase-1/*β*-actin. (e, f) show representative blots of Iba1 and GFAP and quantification data for Iba1/*β*-actin and GFAP/*β*-actin. Data are presented as the mean ± SEM. ^∗^*P* < 0.05 vs. control, ^#^*P* < 0.05 vs. LPS group.

We evaluated the expression of the NLRP3 inflammasome signaling pathway 24 h after LPS injection. The results showed that the levels of NLRP3, ASC, caspase-1, and cleaved-caspase-1 in the hippocampus were significantly increased 24 h after LPS injection, and PSP significantly inhibited these increases (Figures 5(a)–5(d), *P* < 0.05).

We also measured the expression of Iba1 and GFAP. The expression levels of Iba1 and GFAP in the hippocampus were significantly increased 24 h after LPS injection compared with control, and PSP significantly reduced the expression of Iba1 (Figures 5(e) and 5(f) vs. LPS, *P* < 0.05). These results suggested that LPS-induced activation of the NLRP3 inflammasome signaling pathway and glial cells could be inhibited by PSP.

### 3.5. NAC Reduced Oxidative Stress Levels and Inhibited Calpain-Mediated Inflammatory Pathways

**Figure 6:**
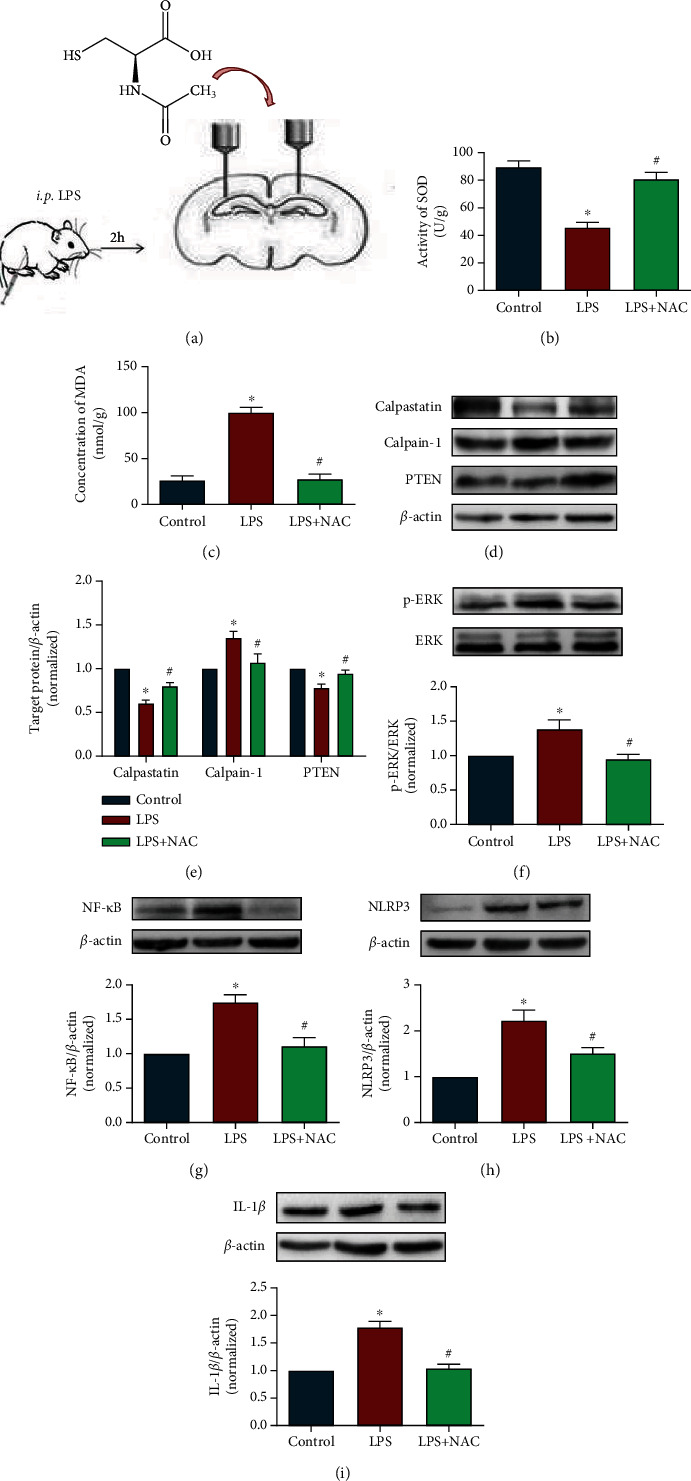
NAC reduced oxidative stress levels and inhibited calpain-mediated inflammatory pathways. (a) shows the schematic design. (b, c) show SOD and MDA levels in the hippocampus. (d) shows representative blots of calpain-1, calpastatin, and PTEN. (e) shows quantification data for calpain-1/*β*-actin, calpastatin/*β*-actin, and PTEN/*β*-actin in experiment 3. (f–i) show representative blots of p-ERK, NF-*κ*B, NLRP3, and IL-1*β* and quantification data for p-ERK/*β*-actin, NF-*κ*B/*β*-actin, NLRP3/*β*-actin, and IL-1*β*/*β*-actin in experiment 3. Data are presented as the mean ± SEM. ^∗^*P* < 0.05 vs. control, ^#^*P* < 0.05 vs. LPS group.

NAC, an antioxidant, was administered prior to evaluation of oxidative stress in the hippocampus (Figure 6(a)). Treatment with LPS resulted in significantly decreased SOD activity and significantly increased MDA levels compared with those in the control group. Treatment with NAC significantly increased SOD activity and decreased MDA level compared with those in the LPS group (Figures 6(b) and 6(c), *P* < 0.05). These results suggested that NAC could ameliorate LPS-induced oxidative damage in the hippocampus. In addition, NAC significantly inhibited the LPS-induced increases in calpain-1 expression, decreases in calpastatin expression, and degradation of PTEN (Figures 6(d) and 6(e), *P* < 0.05).

We also detected the expression of inflammatory pathway-related proteins and inflammatory factors. The results showed that NAC significantly decreased ERK phosphorylation, and the expression levels of NF-*κ*B, NLRP3, and IL-1*β* compared with those in the LPS group (Figures 6(f)–6(i), *P* < 0.05). These results indicated that NAC administration ameliorated oxidative damage, calpain-1 expression, and inflammatory reactions in the brains of mice treated with LPS.

### 3.6. PSP, Calpeptin, MCC, and NAC Reduced LPS-Induced Cytokine Release

**Figure 7:**
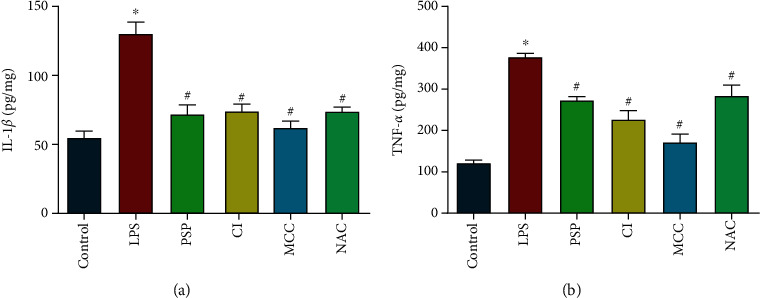
PSP, calpeptin, MCC, and NAC reduced LPS-induced cytokine release. (a, b) show IL-1*β* and TNF-*α* levels in mouse hippocampi. Data are presented as the mean ± SEM. ^∗^*P* < 0.05 vs. control, ^#^*P* < 0.05 vs. LPS group.

We examined the levels of inflammatory cytokine release in the hippocampi of mice in the control, LPS, LPS+PSP, LPS+calpeptin, LPS+MCC, and LPS+NAC groups. The results showed that PSP, calpeptin, MCC, and NAC all significantly reduced the levels of IL-1*β* and TNF-*α* compared with those in the LPS group (Figures 7(a) and 7(b), *P* < 0.05).

### 3.7. PSP Alleviated Depression-Like Behaviors in Animals Subjected to CUMS

**Figure 8:**
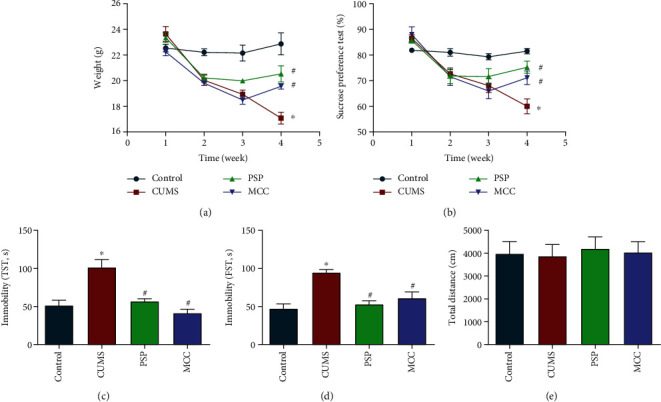
PSP alleviated depression-like behavior in animals subjected to CUMS. (a) shows body weight. (b) shows sucrose preference index changes. (c, d) show the immobility times in the TST and FST. (e) shows the total distance travelled in the OFT. Data are presented as the mean ± SEM. ^∗^*P* < 0.05 vs. control, ^#^*P* < 0.05 vs. CUMS group.

During the modeling process, body weights and sucrose preference indices of the animals subjected to CUMS showed downward trends. At the end of modeling, the body weights and sucrose preference indices of the animals subjected to CUMS were significantly lower than those in the control group. Administration of PSP or MCC administration resulted in significantly increased body weight compared with that in the animals subjected to CUMS (Figures 8(a) and 8(b), *P* < 0.05). The results of the TST and FST tests showed that the immobility times in the CUMS group were significantly higher than those in the control group, and PSP and MCC significantly attenuated this effect (Figures 8(c) and 8(d), *P* < 0.05). No significant differences were observed among the groups in the OFT (Figure 8(e), *P* > 0.05), which indicated that movement activity was not affected.

### 3.8. PSP Regulated Calpain System, Nrf2, and NLRP3 Signaling Pathway in Animals Subjected to CUMS

**Figure 9:**
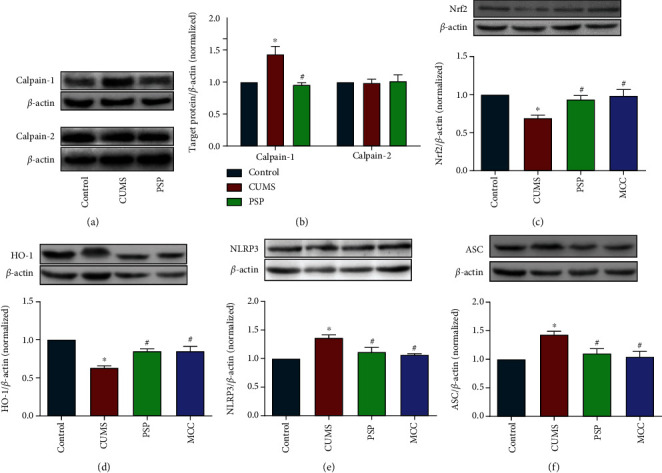
PSP regulated the calpain system and the Nrf2 and NLRP3 pathways in animals subjected to CUMS. (a, b) show representative blots of calpain-1 and calpain-2 and quantification data for calpain-1/*β*-actin and calpain-2/*β*-actin. (c–f) show representative blots of Nrf2, HO-1, NLRP3, and ASC and quantification data for Nrf2/*β*-actin, HO-1/*β*-actin, NLRP3/*β*-actin, and ASC/*β*-actin in animals subjected to CUMS. Data are presented as the mean ± SEM. ^∗^*P* < 0.05 vs. control, ^#^*P* < 0.05 vs. CUMS group.

CUMS significantly increased the expression of calpain-1 but did not affect the expression of calpain-2. Administration of PSP significantly reduced CUMS-induced increases in calpain-1 and NF-*κ*B expression (Figures 9(a) and 9(b), *P* < 0.05). Furthermore, CUMS significantly increased the expression of NLRP3 and ASC and reduced the expression of Nrf2 and HO-1. These changes were reversed by administration of PSP (Figures 9(c)–9(f), *P* < 0.05).

## 4. Discussion

In this study, we showed that LPS treatment increased calpain-1 expression and substrate degradation, activated the NLRP3 inflammasome signaling pathway and glial cells, and reduced Nrf2 and calpastatin expression. Treatment with PSP prevented these LPS-induced changes. In addition, calpeptin, MCC, and NAC inhibited LPS-induced release of proinflammatory cytokines. The potential protective mechanisms of PSP were further confirmed using a CUMS model.

Impairment of the antioxidant system in the brain exacerbates depressive symptoms, and increased oxidative stress has been observed previously in CUMS models [27, 28]. Furthermore, ROS have also been shown to trigger activation of the NLRP3 inflammasome [29]. In this study, we found that LPS increased the cytoplasmic ROS levels in HT-22 cells. Treatment with PSP inhibited LPS-induced production of cytoplasmic ROS in HT-22 cells. The levels of SOD and MDA are markers of ROS-induced oxidative damage [30]. Levels of MDA were increased, and levels of SOD were decreased in the hippocampi in animals treated with LPS, which indicated that LPS induced oxidative stress in the brain. The ROS scavenger NAC reduced the MDA levels and increased SOD levels, which indicated that NAC ameliorated oxidative damage. Our previous study also showed that PSP reduced MDA levels and increased SOD levels [20]. In the cellular experiment, the ROS level could be easily detected using the flow cytometer method with the DCFH-DA staining. By contrast, the ROS level would be damaged during the collection of the hippocampal tissues. Therefore, we measured the MDA and SOD levels in the hippocampal tissues instead. The consistent results from *in vivo* and *in vitro* experiments suggest that PSP could prevent oxidative damage.

The Nrf2/HO-1 axis is the primary pathway involved in the antioxidant response to external stimuli [31]. In addition, the Nrf2/HO-1 signaling pathway is the main sensor of oxidative stress [32]. Continuous oxidative stress is an important contributor to susceptibility to depression. Antioxidants can activate the Nrf2 system, resulting in activation of antioxidant defense mechanisms and reduced susceptibility to depression [6]. Several studies have found that Nrf2 was involved in mitigation of NLRP3 overactivation, which resulted in decreased neuroinflammation [33, 34]. Due to its key role in regulating the cellular antioxidant and anti-inflammatory responses, upregulation of Nrf2 is a common therapeutic target for neuropsychiatric disorders such as major depression, bipolar disorder, and schizophrenia [35]. Our study found that Nrf2 expression was decreased in the hippocampi of animals treated with LPS or subjected to CUMS, and this decrease was prevented by PSP. These results further suggested that PSP could improve oxidative damage and reduce susceptibility to depression by promoting Nrf2 expression. The expression of HO-1 was significantly decreased at 2 h and 6 h after LPS administration, then returned to baseline. However, the expression trends of Nrf2 and HO-1 should be consistent, as Nrf2 is a regulator of HO-1 expression [32]. This discrepancy requires further investigation.

Calpain is closely associated with synaptic plasticity and neurodegeneration and is a key target for many neurological diseases, including Alzheimer's disease and Parkinson's disease [36, 37]. The expression of SCOP is largely limited to the central nervous system, and SCOP is a degradation substrate of calpain. Studies have shown that SCOP regulates long-term memory and negatively correlates with ERK phosphorylation [38]. In addition, PTEN is a degradation substrate of calpain and is associated with the inflammatory response [39]. Calpain activity is mainly regulated by calcium and calpain inhibitors. Calpastatin is a member of the calprotease family, which is a multifunctional endogenous inhibitor of calpain [11]. Therefore, there is a negative regulatory relationship between calpastatin and calpain. Our previous study showed that activation of calpain was closely related to initiation of neuroinflammation, and inhibition of calpain improved depression-like behaviors by reducing degradation of its substrates, inhibiting downstream inflammatory activity [14]. In this study, LPS induced calpain-1 expression, promoted degradation of SCOP and PTEN, and inhibited calpastatin expression. Treatment with PSP inhibited the expression of calpain-1 and promoted the expression of calpastatin. This suggested that PSP could exert antidepressant effects by modulating the calpain system.

The NLRP3 inflammasome is closely associated with depression and neuroinflammation [40]. Therefore, the NLRP3 system may be a key mediator of the association between depression and neuroinflammation. Maturation and release of the inflammatory cytokine IL-1*β* is regulated in part by NLRP3. Assembly of inflammasome protein complexes leads to activation and secretion of proinflammatory cytokines in response to various stress factors [41]. Activation of the NLRP3 inflammasome has been found to be associated with LPS- or stress-induced depression-like behaviors in animals [42, 43]. Activation of NLRP3 has also been observed in patients with major depression [9]. Activation of NLRP3 induces isomer formation or aggregation of ASC, resulting in activation of caspase-1 [44]. Active caspase-1 cleaves IL-1*β* to mature IL-1*β*, which is then released into the extracellular environment. Activation of the NLRP3 inflammasome also promotes release of cytokines and chemokines and microglial activation [45]. The NLRP3/ASC/caspase-1 system plays a key role in neuroinflammation in depression, and inhibition of the NLRP3/ASC/caspase-1 system reduces the expression of proinflammatory cytokines and inhibits activation of microglia [46]. Astrocyte activation, as indicated by expression of GFAP, is closely related to inflammation in the nervous system [47]. In this study, treatment with LPS resulted in increased expression levels of NLRP3, ASC, caspase-1, cleaved caspase-1, and GFAP expression and promoted microglia activation. In contrast, PSP inhibited LPS-induced increases in expression of NLRP3, ASC, caspase-1, cleaved-caspase-1, and GFAP and inhibited microglial activation. Taken together, these results suggested that PSP could regulate the NLRP3 system, which may represent a mechanism of the antidepressant effects of PSP.

**Figure 10:**
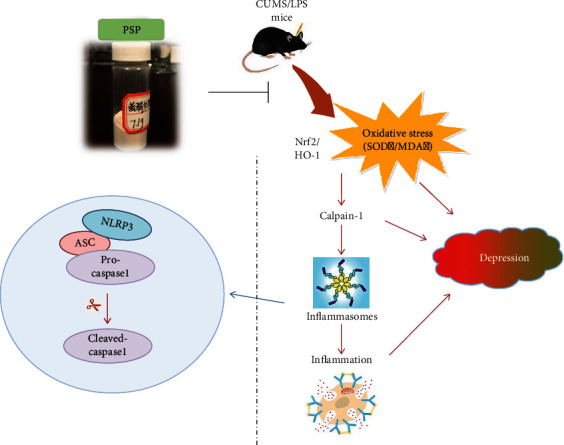
PSP exerts antidepressant effects by regulating the oxidative stress-calpain-1-neuroinflammation signaling axis. Calpain-1 may bridge inflammation and oxidative stress, two relatively independent mechanisms of depression, while PSP plays an antidepressant effect by regulating the signaling axis of oxidative stress-calpain-1-neuroinflammation.

Our previous study found that PSP exerted antidepressant effects by reducing inflammation, oxidative stress, serum cortisol levels, and synaptic and cellular damage [20]. Oxidative stress and inflammation are important interrelated mechanisms in depression. In a clinical study of patients with major depression, markers of oxidative stress and inflammation were elevated [48]. Furthermore, inflammation may induce oxidative damage [49, 50]. A study showed that Nrf2 ameliorated early brain injury associated with intracerebral hemorrhage through inhibition of the NF-*κ*B pathway and NLRP3 inflammasome activation [51]. Increased oxidative stress and excitatory toxicity can promote activation of the calpain system, which can contribute to onset and progression of numerous diseases [15]. Calpain activation induced by elevated ROS levels may play an important role in depression-like behaviors induced by chronic stress [52]. This finding suggests that oxidative stress-induced inflammation might be closely related to the activation of calpain, and the increased oxidative stress may result in abnormal activation of calpain and increased inflammatory response. In this study, intracerebral administration of NAC, an antioxidant, inhibited calpain-1 expression, PTEN degradation, and activation of NF-*κ*B and the NLRP3 inflammasome. These results suggested that calpain may bridge inflammation and oxidative stress, two relatively independent processes associated with depression. We administered PSP, calpeptin, NAC, or MCC to LPS-treated animals and showed that each of these agents blocked the downstream inflammatory response, which indicated that PSP may play an antidepressant effect by regulating the oxidative stress-calpain-1-neuroinflammation signaling axis (Figure 10).

Administration of LPS is an acute stress model, CUMS is a chronic stress model, and neuroinflammation occurs in both models [53]. In the CUMS model, PSP improved depression-like behaviors and inhibited the expression of calpain-1 and inflammation-related proteins. This further confirmed that PSP might exert antidepressant effects through this signaling axis. We did not investigate the effect of molecular weight distribution on PSP antidepressant efficacy. Future studies should evaluate the effects of monosaccharide composition and molecular weight distribution on the antidepressant effects of PSP to characterize the structure-activity relationship. In addition, the role of calpain in the pathogenesis of depression should be evaluated in multiple cell types.

In conclusion, this study indicates that PSP exerts antidepressant effects in mice via the oxidative stress-calpain-1-NLRP3 signaling axis. This study suggests that PS may be an important nutrient for the treatment of depression.

## Data Availability

The datasets used and/or analyzed during the current study are available from the corresponding author on reasonable request.

## Supplementary Materials

Supplementary Materials sFig. 1: qualitative and quantitative analysis of PSP. sFig. 2: PSP alleviates LPS-induced depression-like behaviors in LPS mice. sFig. 3: PSP ameliorates LPS-induced oxidative stress.
