# Supplementary material for: Polysaccharides from Polygonatum cyrtonema Hua Reduce Depression-Like Behavior in Mice by Inhibiting Oxidative Stress-Calpain-1-NLRP3 Signaling Axis
Source: Oxid Med Cell Longev. 2022 Apr 20;2022:2566917. doi: 10.1155/2022/2566917 (PMC9045988; doi:10.1155/2022/2566917)
Supplement: Supplementary Materials — sFig. 1: qualitative and quantitative analysis of PSP. sFig. 2: PSP alleviates LPS-induced depression-like behaviors in LPS mice. sFig. 3: PSP ameliorates LPS-induced oxidative stress. [file 2566917.f1.docx]

**Supplementary Materials for**

**Inhibition of the oxidative stress-calpain-1-NLRP3 signaling axis by polysaccharides from polygonatum cyrtonema Hua reduced depression-like behaviors in mice**

**The PDF file includes:**

**sFigs.1 to 3**

**Supplementary figures and captions**


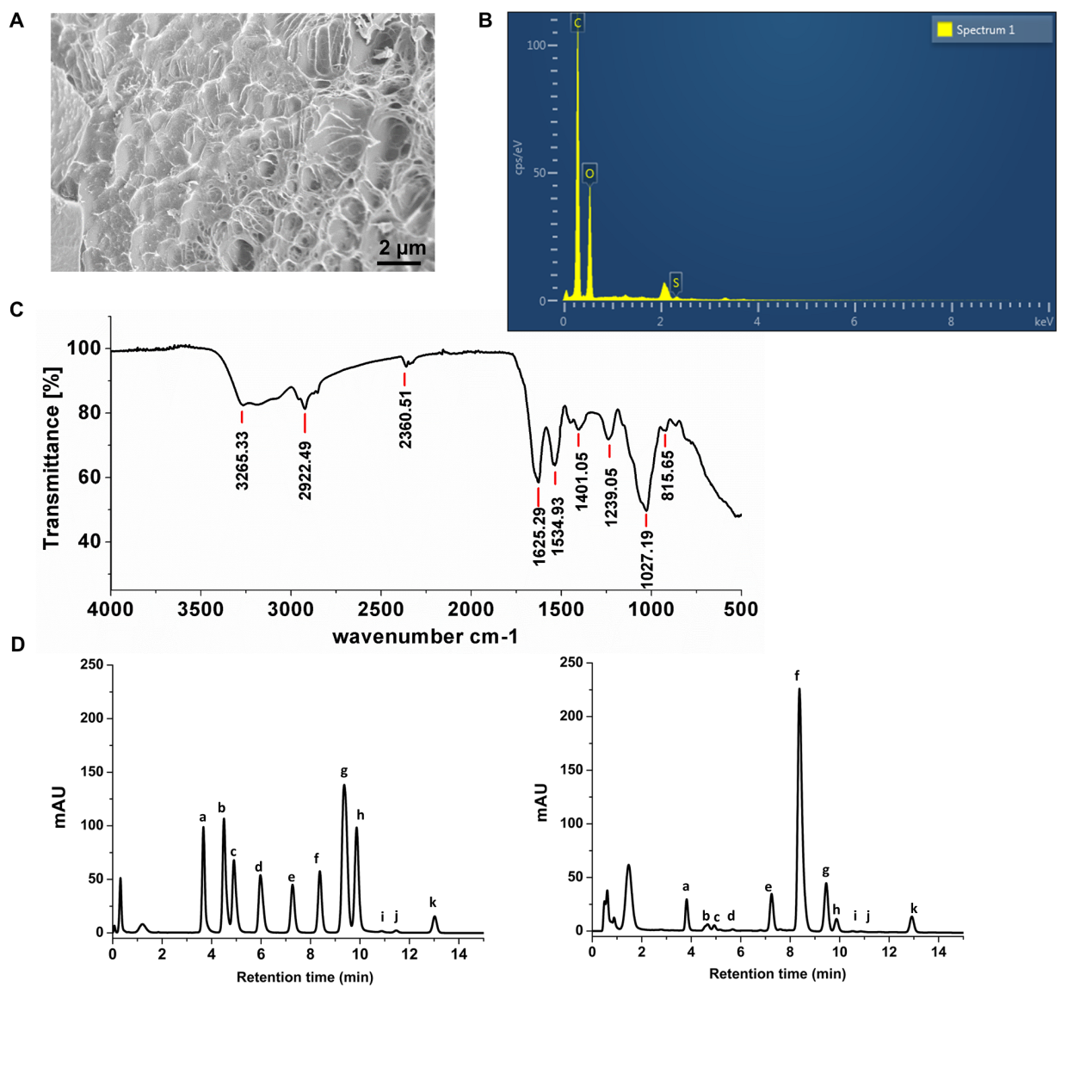


**sFig.1.** Qualitative and quantitative analysis of PSP. (A) SEM images of PSP. Scale bar is 2 μm; (B) Energy spectrum of PSP. (C) Infrared spectra of PSP; (D) The UHPLC chromatograms of PMP derivatives of standard monosaccharide mixture and monosaccharides released from PSP (a. Mannose, tR=3.84 min; b. Ribose, tR=4.58 min; c. Rhamnose, tR=5.03 min; d. Glucuronic acid, tR=5.91 min; e. Galacturonic acid, tR=7.33 min; f. Glucose, tR=8.39 min; g. Galactose, tR=9.31 min; h. Arabinose, tR=9.76 min.)


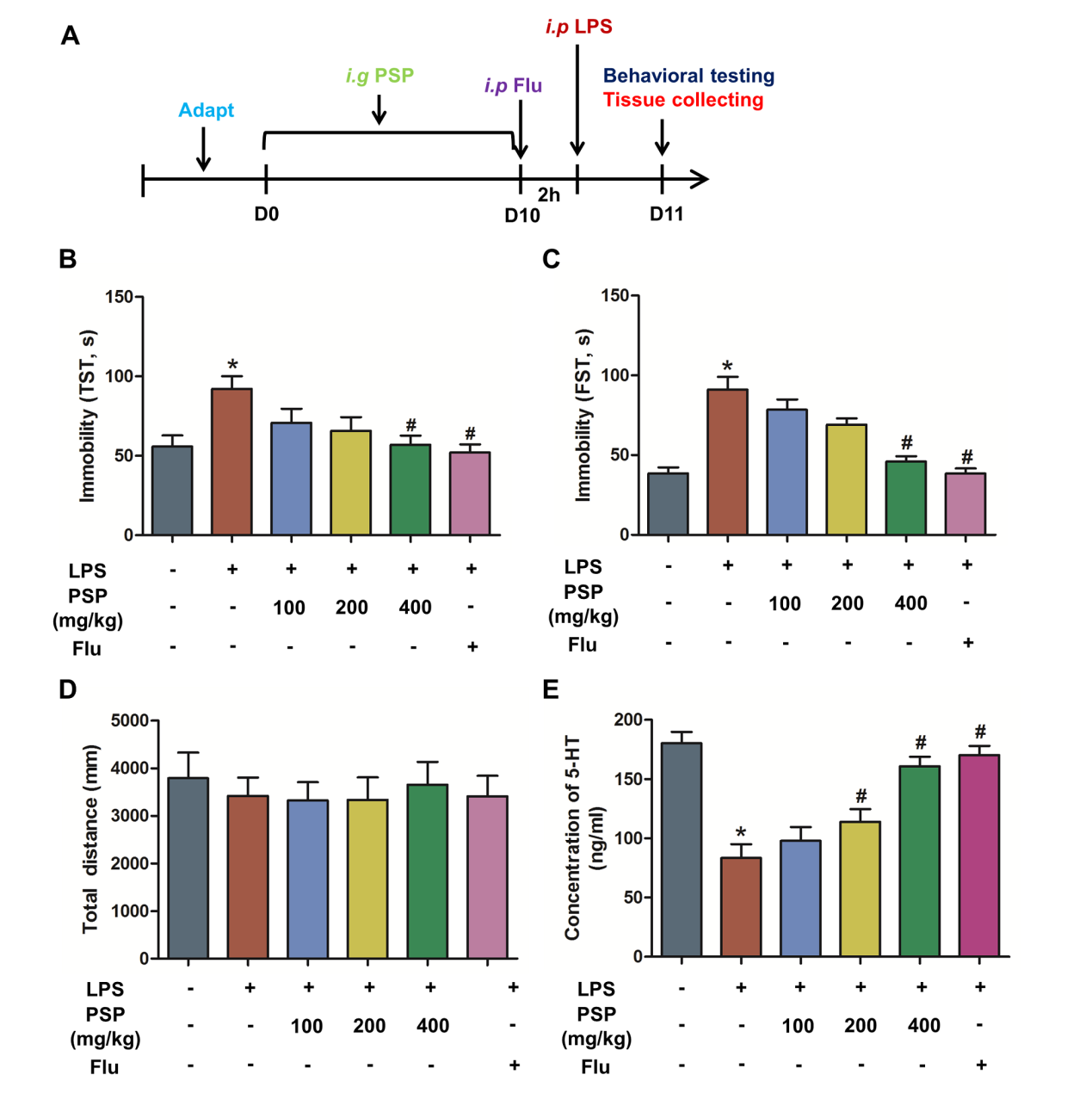


**sFig.2.** PSP alleviates LPS-induced depression-like behaviors in LPS mice (n=8). (A) Schematic illustrating the study design (n=8); (B) The tail suspension test (n=8); (C) The forced swimming test (n=8); (D) The open field test (n=8). (E) ELISA detecting 5-HT level in the hippocampus (n=6). The results are presented as the mean ± SEM. ^*^*P* < 0.05 vs Control, ^#^*P* < 0.05 vs LPS group.

**
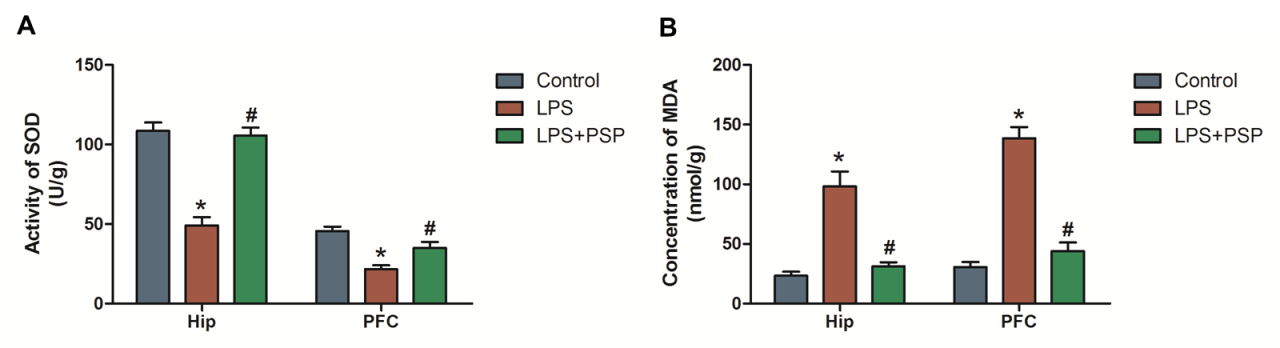
**

**sFig.3.** PSP ameliorates LPS-induced oxidative stress. (A) SOD level in the hippocampus and prefrontal cortex (n=6). (B) MDA level in the hippocampus and prefrontal cortex (n=6). Data are presented as mean ± SEM. ^*^*P* < 0.05 vs Control, ^#^*P* < 0.05 vs LPS group.
